# Present Practice of Radiative Deep Hyperthermia in Combination with Radiotherapy in Switzerland

**DOI:** 10.3390/cancers14051175

**Published:** 2022-02-24

**Authors:** Emanuel Stutz, Emsad Puric, Adela Ademaj, Arnaud Künzi, Reinhardt Krcek, Olaf Timm, Dietmar Marder, Markus Notter, Susanne Rogers, Stephan Bodis, Oliver Riesterer

**Affiliations:** 1 Department of Radiation Oncology, Inselspital, Bern University Hospital, University of Bern, 3010 Bern, Switzerland; emanuel.stutz@insel.ch (E.S.); reinhardt.krcek@insel.ch (R.K.); 2 Department of Radiation Oncology KSA-KSB, Kantonsspital Aarau, 5001 Aarau, Switzerland; emsad.puric@ksa.ch (E.P.); adela.ademaj@ksa.ch (A.A.); olaf.timm@ksa.ch (O.T.); dietmar.marder@ksa.ch (D.M.); susanne.rogers@ksa.ch (S.R.); s.bodis@bluewin.ch (S.B.); 3 Doctoral Clinical Science Program, Medical Faculty, University of Zürich, 8032 Zürich, Switzerland; 4 Clinical Trials Unit, University of Bern, 3010 Bern, Switzerland; arnaud.kuenzi@ctu.unibe.ch; 5 Radiation Oncology, Lindenhofspital Bern, 3012 Bern, Switzerland; markus.notter@lindenhofgruppe.ch; 6 Department of Radiation Oncology, University Hospital Zurich, 8032 Zürich, Switzerland; 7 Foundation for Research on Information Technologies in Society (IT’IS), 8004 Zürich, Switzerland

**Keywords:** moderate hyperthermia, deep hyperthermia, radiative hyperthermia, radiotherapy, patterns of care, reimbursement

## Abstract

**Simple Summary:**

Moderate hyperthermia is a potent radiosensitizer and its efficacy has been proven in randomized clinical trials for specific tumor entities. In spite of this, hyperthermia still lacks general acceptance in the oncological community and implementation of hyperthermia in clinical practice is still low. Reimbursement is one key factor regarding the availability of hyperthermia for deep-seated tumors, with high variability in reimbursement between countries. We report the current reimbursement status and related pattern of care for the use of deep hyperthermia in Switzerland over a time period of 4.5 years. This analysis will provide the basis for the national standardization of deep hyperthermia treatment schedules and quality assurance guidelines, as well as for the expansion of deep hyperthermia indications in the future. This comprehensive insight into deep hyperthermia reimbursement and practice in Switzerland might also be of interest for other national hyperthermia societies.

**Abstract:**

Background: Moderate hyperthermia is a potent and evidence-based radiosensitizer. Several indications are reimbursed for the combination of deep hyperthermia with radiotherapy (dHT+RT). We evaluated the current practice of dHT+RT in Switzerland. Methods: All indications presented to the national hyperthermia tumor board for dHT between January 2017 and June 2021 were evaluated and treatment schedules were analyzed using descriptive statistics. Results: Of 183 patients presented at the hyperthermia tumor board, 71.6% were accepted and 54.1% (99/183) finally received dHT. The most commonly reimbursed dHT indications were “local recurrence and compression” (20%), rectal (14.7%) and bladder (13.7%) cancer, respectively. For 25.3% of patients, an individual request for insurance cover was necessary. 47.4% of patients were treated with curative intent; 36.8% were in-house patients and 63.2% were referred from other hospitals. Conclusions: Approximately two thirds of patients were referred for dHT+RT from external hospitals, indicating a general demand for dHT in Switzerland. The patterns of care were diverse with respect to treatment indication. To the best of our knowledge, this study shows for the first time the pattern of care in a national cohort treated with dHT+RT. This insight will serve as the basis for a national strategy to evaluate and expand the evidence for dHT.

## 1. Introduction

Moderate-temperature (39–45 degree Celsius) regional hyperthermia (HT) is concurrently applied with radiotherapy (RT) or chemotherapy [1]. Adding HT to RT improves treatment outcomes such as local tumor control or overall survival in specific tumor entities with a negligible toxicity profile [2,3]. HT can be applied with superficial HT devices for superficial tumors (less than 4 cm depth below the skin) or with deep HT (dHT) devices for tumors located at depth (more than 4 cm from the skin). Several techniques and devices for the clinical application of dHT exist [1,4,5]. Although its effect has been proven in several tumor entities with positive phase III randomized trials and meta-analyses [3], there is no widespread use in Europe. Reasons are multifactorial and have been previously summarized by Van der Zee et al. [1] and Overgaard et al. [6], but are still ”hot”. Briefly, not only proving that the tumor region was adequately heated but also to heat and sustain a uniform temperature in the tumor region are challenging as the body attempts to maintain temperature homeostasis. Some earlier trials with dHT reported questionable results with worse outcomes with dHT, most probably caused by insufficient heating, missing quality assurance and an imbalance in the patient groups ([7] and discussion in [8]). This confusion resulted in a persistent loss of credibility in the oncological community [6,8,9].

Another reason for the lack of widespread availability is that HT, and especially dHT, is relatively labor-intensive and needs trained staff [1,10]. Furthermore, the use of dHT as a radiosensitizer competes with concurrent chemotherapy. The advantages of chemotherapy include easy administration, a lesser requirement of technical experience and comprehensive availability. The prime example of this is cervical cancer ([11], discussion in [12]). A financial obstacle is the uncertain cost reimbursement of HT treatment in most countries, limiting HT practice to university centers [8,9] and withholding it from the broader target population. Therefore, despite good but aged evidence, only a few dHT indications were incorporated into international oncology treatment guidelines.

HT has a long tradition in Switzerland, starting in 1980 with the first clinical application of superficial HT with RT at the Center for Radiation-Oncology Kantonsspital Aarau. In 1988, the first dHT treatment in combination with RT (dHT+RT) was performed there. Superficial HT was later rolled out to a second hospital in Switzerland and clinical applications, mainly for recurrent breast cancer, were maintained at this site. Thus, prior to 2017, there were only two centers applying HT based on ESHO guidelines [13,14,15,16] in Switzerland (Kantonsspital Aarau and Lindenhofspital Bern), with only the Kantonsspital Aarau applying dHT. During this time, for every HT treatment, an individual request to the patients’ health insurance for reimbursement was required. The national Swiss Hyperthermia Network (SHN) was founded to synchronize and coordinate HT research activities at the national level, guarantee treatment quality and improve the evidence base for HT. In 2016, the SHN submitted a proposal for the reimbursement of HT+RT for selected evidence-based indications to the Swiss Federal Office of Public Health for superficial HT and dHT. Subsequently, four indications for superficial HT and five indications for dHT were temporarily approved for reimbursement for a period of two years as from 2017 (Table 1). It was stipulated that every patient receiving HT had to be presented to and have the indication confirmed by the national SHN tumor board, which was constituted by HT experts to guarantee the high quality of treatment decisions [17,18,19,20]. For patients who were likely to benefit from dHT+RT without a listed reimbursed indication, a specific request for insurance cover was necessary.

At the end of the 2 years, the SHN submitted an update of the current evidence for dHT to the Swiss Federal Office of Public Health. After reevaluation, dHT indications were expanded in 2019 with the indications of “local tumor recurrence and compression” and “painful bone metastasis”, making a total of seven reimbursed dHT indications. As of July 2021, the Swiss Federal Office of Public Health granted unrestricted coverage for the dHT indications of “cervical cancer” and “painful bone metastasis”. Reimbursement for the dHT indications “local tumor recurrence and compression” and “soft tissue sarcoma” has been temporarily prolonged, again for another 2-year time period. The indications for bladder, pancreatic and rectal cancer lost their reimbursement status (Table 1) [20].

Regarding superficial HT, four indications (specific situations in breast and head and neck cancer, malignant melanoma and palliative indications with local tumor compression), were granted for two years and then without time restrictions [17,19]. However, superficial HT is not within the scope of the present analysis.

To the best of our knowledge, this is the first analysis of an unselected, dHT patient cohort regarding treatment indications, patient and tumor characteristics and treatment schedules. We aimed to perform a pattern of care analysis to shed more light on dHT practice in Switzerland and build a basis for a national strategy to evaluate, consolidate and expand the evidence for dHT.

## 2. Materials and Methods

All patients presented at the SHN tumor board between January 2017 and June 2021 for the evaluation of radiative dHT+RT based on ESHO guidelines [13,14] were collected in a database. In July 2021, the reimbursed dHT indications changed and, since the end of 2021, a second center in Switzerland has started to apply dHT. This time period included a patient cohort treated by a single dHT center with only one modification of reimbursed dHT indications.

Data from tumor board protocols were independently extracted and crosschecked by two authors regarding reimbursed dHT indications, patient and tumor characteristics and information regarding referring hospitals. These data then were crosschecked and completed with dHT and RT treatment details by three other authors. In case of any discrepancy, a consensus was reached. This project was approved by the local ethics committee (EKNZ2021-01022, 1 July 2021).

Possible candidates for dHT were presented at the weekly national SHN tumor board by their referring physicians. The individual indication for dHT was discussed with at least two radiation oncologists with clinical experience in moderate dHT, including also senior medical oncologists. Indications were approved if the patient exhibited no contraindications for dHT (e.g., metal implant, cardio-pulmonary insufficiency, etc.), if dHT was technically feasible (only treatable lesions in accessible tumor locations) and if there was no other more appropriate treatment option (i.e., RT alone, hormone therapy, chemotherapy or immunotherapy).

### 2.1. Principles of Application of Deep Hyperthermia

From 2017 to 2021, Kantonsspital Aarau was the only institution providing radiative dHT+RT in accordance with ESHO guidelines [13,14] and therefore received referrals from centers throughout Switzerland. Not only the optimal treatment sequence of HT and RT but also the optimal time interval between RT and HT or vice versa is still a matter of debate. Multiple working mechanisms requiring different optimal temperature ranges contribute to the effectiveness of HT, as comprehensively presented in Oei et al. [40]. In the absence of robust clinical data, the decision on the therapeutic sequence of HT and RT is made individually by the respective center. Preclinical studies indicated that the time interval between RT and HT should be kept as short as possible [41] but clinical studies addressing the time interval are sparse [42,43,44,45]. In two retrospective clinical studies investigating the effect of the time interval on treatment outcomes in cervical cancer patients, one revealed a strong correlation of a short time interval between RT and dHT for a better clinical outcome [44], where the other study showed that a time interval up to 4 h has no effect [45]. These contradicting results initiated a comprehensive discussion that depicted the complexity of this topic [46,47,48]. However, with regard to the dHT standard operating procedure at the Kantonsspital Aarau, dHT is given before RT with a minimal time interval.

dHT was performed with the BSD 2000 3D Hyperthermia Systems© (BSD Medical Corporation/Pyrexar, Salt Lake City, UT, USA) using either the SigmaEye© or Sigma 60© applicator, depending on the diameter of the abdomen or limb. The interval between two dHT treatments was at least 72 h. For pelvic dHT, thermometry probes were inserted in the bladder, the rectum, the vagina, the anal margin and superficially on both groins for continuous thermometry and thermal mapping where possible/necessary. Interstitial thermometry was not performed except for patients receiving interstitial brachytherapy. For all other patients, the hyperthermia treatment planning software Sigma Hyperplan© (M/s Dr. Sennewald Medizintechnik GmbH, Munich, Germany) was used to estimate suitable power and steering parameters to achieve the targeted tumor temperature of 41 °C. A dHT session starts with a warm-up heating phase. The following plateau phase had a duration of 60 min and started when (a) the targeted temperature in the tumor was reached (this option was only possible if the heated tumor was adjacent to an intraluminal thermometry probe), (b) the targeted power and steering parameters were reached or (c) latest after a 30 min warm-up heating phase, respectively. During treatment, vital functions were continuously monitored.

The frequency of dHT was determined individually. Usually, dHT once per week was used for curative indications and dHT twice per week for palliative indications. 

As not every patient started RT on a Monday, a reliable subdivision of dHT once versus twice per week was not possible. For the purpose of this study, dHT frequency was therefore categorized as once or once to twice a week. For patients referred from other hospitals, the optimal RT schedule in combination with dHT was discussed at the SHN tumor board; however, the final responsibility for the RT schedule lay with the referring center. Whenever possible, patients were treated within or analogous to an existing treatment protocol. 

Some patients treated for bladder, rectal, anal and pancreatic cancer received a trimodal treatment with dHT+RT and concurrent chemotherapy. These patients were treated within [49,50,51] or analogous to a clinical trial [50,51,52,53,54]. Patients were divided into “in-house” and “referred” patients. Every patient originating from the Kantonsspital Aarau was considered “in-house”. Additionally, patients from other hospitals without RT facilities, which referred patients for RT to the Kantonsspital Aarau, were also considered “in-house”. Patients from other hospitals with RT facilities who were referred for dHT were classified as “referred patients”, independent of where they finally received the RT treatment. To depict the spatial policy of referrals, referring hospitals were further divided into intra-cantonal and extra-cantonal and the distance by road from the referring hospitals to the Kantonsspital Aarau was calculated. There were three options for the organization of the dHT+RT treatment: (1) the patient received both dHT+RT at the Kantonsspital Aarau, (2) the patient received RT at the day of the dHT session at the Kantonsspital Aarau and the remainder of the RT at the referring hospital or (3) the patient received dHT sessions only at Kantonsspital Aarau and all RT sessions at the referring hospital. The latter option was deemed suboptimal based on the standard operating procedure at the Kantonsspital Aarau, wherein dHT should be given before RT with a minimal time interval. If not possible, a latency of 90 min between HT and RT was deemed acceptable. For patients treated with protons at the Paul Scherrer Institute, only option 3 was possible; however, the distance by road was less than 30 km. For referred patients, option 2 was preferred due to the short latency between RT and dHT. During the COVID-19 pandemic, this option was omitted to avoid mixing in-house and external patients to decrease the risk of infection. The time interval between dHT and start of the following RT was measured in patients receiving both dHT and RT at the Kantonsspital Aarau and was defined as the time between switching power off on the dHT device and first beam-on of the RT. Time points were extracted from automatical treatment recordings and stated in minutes.

### 2.2. Statistics

Descriptive statistics were used to describe patient and tumor characteristics and treatment details, which were presented as mean with standard error, median with (interquartile) range or frequencies with percentages, depending on their distribution. 

Data were represented using Statistical Package R (released 2021, 10 August, Version 4.1.1) and the ggplot2 package, version 3.3.5. Due to the combination of the small sample size, many stratification levels and wide heterogeneity of treatment and patient characteristics, statistical inference was not performed beyond the summary tables presented here as it was judged that a qualitative assessment of the data would be more suited to the aims of this study. Continuous values were summarized with mean, standard deviation, median and max/min values. Categorical variables were summarized as frequencies and proportions.

The river plot was generated using the free, internet-based software SankeyMATIC [55].

## 3. Results

### 3.1. Patient Flow through the Swiss Hyperthermia Network Tumor Board

**Figure 1:**
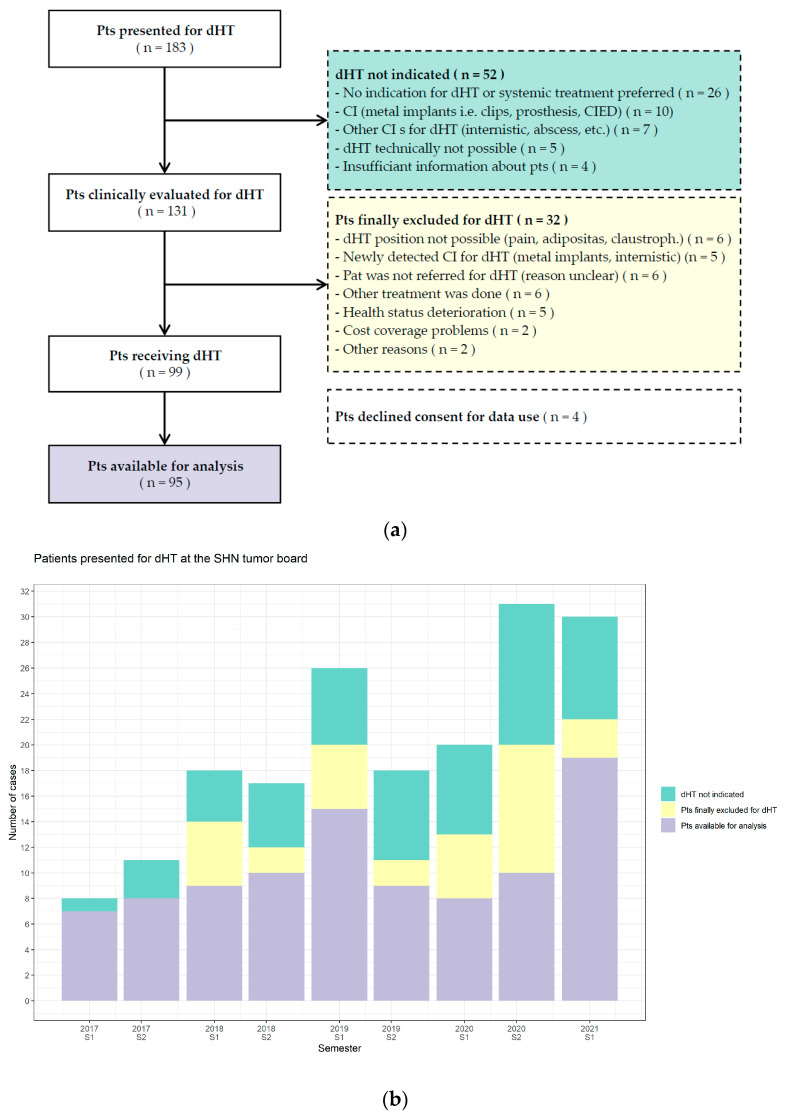
Patient flow through the SHN tumor board. (**a**) Patients presented for dHT were excluded if dHT was not indicated (green) or a physical examination and treatment tolerability check revealed an exclusion criterion (yellow). Only patients with informed consent were eligible for analysis (violet). Background colors match the corresponding bar chart plot. (**b**) Patients presented at the SHN tumor board from January 2017 to June 2021 were depicted per semester. Events that may have affected the number of patients and indications treated were the two new “reimbursed dHT indications” as of 2019 and the changes in oncological treatment patterns during the COVID-19 pandemic, especially the COVID-19 lockdown in Switzerland (11 March to 26 April 2020; 1st semester 2020). Abbreviations: CI: contraindication, CIED: cardiac implantable electronic device, Claustroph: claustrophobia, dHT: deep hyperthermia, Sem: semester, SHN: Swiss Hyperthermia Network, Pts: patients, S1: 1st semester, S2: 2nd semester.

Between January 2017 and June 2021, 567 patients were presented for the evaluation of superficial or deep hyperthermia, with 32.3% (183/567) qualifying for dHT. Of these 183 patients, 28.4% (52/183) were deemed unsuitable. The remaining 131 patients were further assessed at a medical consultation and by their ability to tolerate the patient positioning required for dHT. This resulted in the further exclusion of 24.4% of patients (32/131). The reasons are stated in Figure 1a. In total, 54.1% (99/183) of patients initially presented at the SHN tumor board actually received dHT. Four patients had to be excluded due to withdrawal of consent, resulting in a total of 95 patients for analysis. Patients for superficial HT were beyond the scope of this analysis.

### 3.2. Patient Characteristics

The median age of patients receiving dHT was 65 years (range, 18–88). Moreover, 57.9% (55/95) of patients were male and 49.5% (47/95); 41.1% (39/95) and 9.5% (9/95) had an Eastern Cooperative Oncology Group (ECOG) performance score of 0, 1 or 2, respectively. A total of 47.4% (45/95) of patients received dHT with curative intent. Meanwhile, 42.1% (40/95) of patients had been previously irradiated and received dHT combined with re-irradiation (re-RT). In addition, 7.4% (7/95), 23.2% (22/95) and 69.5% (66/95) of patients were treated within a study protocol [49,50,51], analogous to a protocol [50,51,52,53,54] or as part of routine clinical practice, respectively (Table 2).

**Figure 2:**
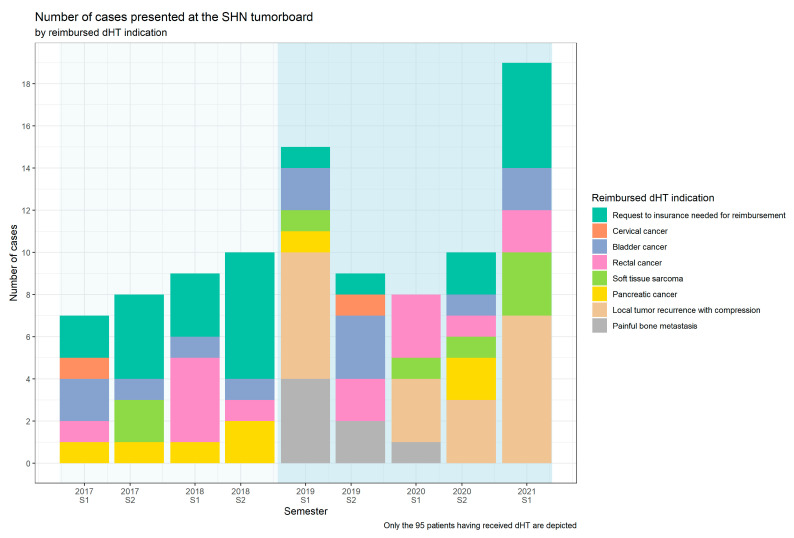
Trend of patients treated with combined deep hyperthermia (dHT) and radiotherapy over time. Bar chart where numbers of patients receiving dHT between January 2017 and June 2021 are depicted per semester (S1 and S2) and divided into “reimbursed dHT indications” with specific subgroups and “request for insurance cover”. From 2017 to 2018, a linear increase in patient numbers with approx. 1 patient per semester was showed. Two new reimbursed indications, “local tumor recurrence with compression” and “painful bone metastasis”, were granted as from 2019 (blue shaded background). COVID-19 lockdown in Switzerland was during 1st semester 2020 (11 March to 26 April 2020).

Patients were divided into groups based on treatment indication regarding reimbursement status (reimbursed dHT indications vs. indication requiring an individual “request for insurance cover”) and based on primary tumor entities, respectively (Table 2, Figure 2, Supplementary Data, Figure S1). This revealed that “local tumor recurrence with compression” was the most common reimbursed dHT indication treated, representing 20.0% (19/95) of patients, followed by “rectal cancer” with 14.7% (14/95) and “bladder cancer” with 13.7% (13/95) of patients. Over the 4.5-year time period, 24.2% of patients (24/95) were treated with an indication not directly covered or not yet covered and therefore required an individual “request for insurance cover” to obtain reimbursement. Details of this patient group are provided in the Supplementary Data in Table S1. 15 of 24 patients who were treated from 2017 to 2018 and therefore before the two new dHT indications (“tumor local recurrence and compression” and “painful bone metastasis”) were added, as well as 9/24 patients in the time period from 2019 to the first semester of 2021. Ten of these 15 patients would have fallen within the two new indications, showing that the two new indications covered an existing demand. 

Regarding primary cancer entities, the most common was rectal cancer, with 22.1% (21/95), followed by bladder cancer with 15.8% (15/95) and soft tissue sarcoma with 13.7% (13/95) of patients (Table 2). Tumor entities with less than three treated patients are not individually represented but summarized in the group “others”, which contributed with 18.9% (18/95). Primary cancer entities, i.e., anal, colon and prostate cancer, presented in a clinical situation belonging to the reimbursed indications “local tumor recurrence and compression”, “painful bone metastasis” or to the group “request for insurance cover”. The time trend is shown in the Supplementary Data, in Figure S1.

The patient population treated with dHT consisted of 36.8% (35/95) in-house and 63.2% (60/95) of patients referred from external radiation oncology institutions. To depict the spatial policy of referrals, the distance from the referring hospital to the Kantonsspital Aarau was calculated, resulting in a mean of 61.5 km (SD 54.3 km) and a median of 42 km (range 23–238 km) (Table 2).

**Figure 3:**
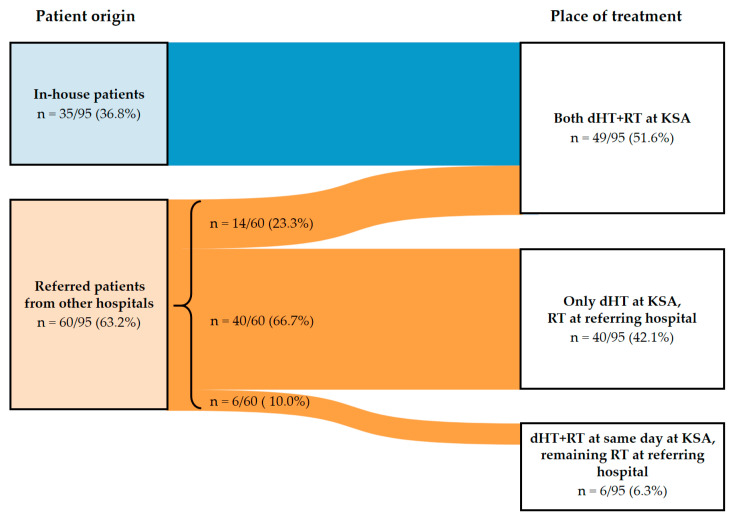
River plot showing the proportions of in-house and referred patients and where the RT and dHT were applied. On the left, patients are grouped according to source of referral. On the right, the three options regarding where and how dHT+RT treatment was applied are stated. The thickness of the connecting flowlines represents the proportion of patients. Abbreviations: dHT: deep hyperthermia, dHT+RT: combined dHT and RT, KSA: Kantonsspital Aarau (dHT center), RT: radiation.

All in-house patients received their RT at the Kantonsspital Aarau. Regarding the patients referred from other hospitals, 23.3% (14/60) of them received both, dHT with all irradiations, at the Kantonsspital Aarau. Moreover, 10.0% (6/60) of patients received all irradiations at their referring hospital except at the day of dHT, where RT was applied at the Kantonsspital Aarau to minimize the time delay between HT and RT. In addition, 66.7% (40/60) of patients received only dHT treatment at the Kantonsspital Aarau and were irradiated at their referring hospital (Figure 3).

Patient characteristics are described more in detail in Supplementary Table S2, comparing (1) in-house vs. referred patients, (2) patients receiving dHT in the setting of a re-RT vs. primary RT, (3) patients treated with palliative vs. curative intention or (4) patients treated within a clinical trial, analogous to a trial or in clinical routine practice, respectively (Supplementary Table S3A). Interestingly, (5) a gender difference was noted (Supplementary Table S4). 

### 3.3. Treatment Characteristics

One of the 95 treated patients stopped dHT+RT after three RT fractions due to reasons unrelated to treatment. This patient was excluded from treatment schedule analysis. In the whole cohort, a mean of 5.24 (SD ± 1.94) and a median of 5 (range 1–10) dHT sessions were applied, with 52.1% (49/94) of patients receiving it once a week and 47.9% (45/94) once to twice a week. Concurrent dHT was applied with external body RT (EBRT), stereotactic body RT (SBRT), protons and interstitial HDR-brachytherapy in 84% (79/94), 2.1% (2/94), 9.6% (9/94) and 4.3% (4/94) of patients, respectively. The mean total number of fractions was 21.7 (SD ± 8.89), with a median of 25 (range 4–38), a mean dose per fraction of 2.49 Gy (SD ± 1.35) and a median of 2 Gy (range 1.8–9 Gy). The mean total dose was 46.2 Gy (SD ± 12.8), with a median of 50 Gy (range 12.5–76 Gy). Moreover, 20.2% (19/94) of patients received an RT boost. RT was delivered daily in 83% (78/94) of patients (Table 3, Supplementary Table S3B). In total, 55 of 95 patients (57.9%) received dHT followed by RT at the Kantonsspital Aarau. The remaining 40 patients travelled to their referring hospital after the dHT session for the same-day RT (Figure 3). In the first group, the time interval between dHT and RT was available in 98.1% of patients (54/55). The mean and median time between the end of the dHT session and start of the RT was 19 min (SD ± 5.5) and 18 min (range 11–32 min), respectively. Evaluation of the time interval of the 40 patients receiving all RT at their referring institution was not possible due to the retrospective nature of this study and because these patients were irradiated at several RT facilities located all over the country. Treatment characteristics were compared between specific patient subgroups, including in-house vs. referred patients, primary RT vs. re-RT and curative vs. palliative intention (Table 3). The treatment schedules employed are stated per dHT indication and per individual patient in detail in Supplementary Table S5.

The specific treatment schedules were dependent on the treatment indication, aim of treatment, pre-irradiation status, primary tumor entity and tumor stage. Patients treated with curative intent generally received a higher total dose, more RT fractions, usually 2 Gy per fraction and one dHT session per week. Palliative or re-RT treatment schedules mostly consisted of lower total doses, less RT fractions using moderate hypofractionation with 1–2 dHT sessions per week, but nearly the same total number of dHT sessions as in the curative setting. This coincides with the expected current practice in radiation oncology.

### 3.4. Hyperthermia Treatment Adherence

The adherence to dHT was high, with 94% (89/95) of patients finishing all dHT sessions as initially prescribed. Six patients did not complete the prescribed sessions. Three of these six patients were treated for bladder cancer, two of them with tetramodal treatment (transurethral resection of bladder tumor (TUR-BT), chemotherapy, dHT+RT) and one with dHT+RT only. The reason for early discontinuation in these three patients was bladder irritation and/or bacterial cystitis, which prevented further catheterization for thermometry. Furthermore, 2/6 patients were treated for rectal cancer with local tumor recurrence with compression with palliative intent and were of ECOG 2. The reason for early discontinuation of dHT was deterioration of health status. The sixth patient was scheduled to receive neoadjuvant dHT+RT for soft tissue sarcoma of the limb. dHT was discontinued after the first HT session due to heat-induced pain in the tumor. 

## 4. Discussion

During the investigated time period, only one RT center in Switzerland provided radiative dHT and seven dHT indications were approved for reimbursement in Switzerland. For other tumor situations that were likely to benefit from combined dHT+RT, an individual request to the patient’s insurance company was necessary. A prerequisite for coverage of the costs stipulated by the Swiss Federal Office of Public Health was the presentation and confirmation of the dHT indication at the SHN tumor board.

Our analysis of the patient flow through this tumor board revealed a high number (approximately 50%) of patients who were not approved for dHT. This might be explained not only by the critical evaluation of the dHT indication by an expert panel, thus reflecting the quality of the tumor board decisions, but also by the fact that some referring physicians were not yet familiar with dHT as they presented patients with obvious contraindications, such as metal implants in the tumor region. We noted that only for two patients dHT could not be applied due to lack of cost recovery (Figure 1a), showing that health insurance companies in Switzerland will cover dHT when no other local treatment options than dHT+RT exist and the indication can be justified. The strict supervision of meaningful indications by the SHN tumor board probably contributed to the high acceptance rate of the health insurers. Therefore, we conclude that the SHN tumor board serves not only for the preselection of patients, besides contributing to the transparency and harmonization of treatment schedules, but also plays a role in teaching newcomers to the field. 

This analysis presents compelling evidence of an existing clinical demand for dHT for both palliative and curative indications. The majority (74.7%, 71/95) of patients in this analysis were treated based on the seven “reimbursed dHT indications” and only 25.3% (24/95) of patients required an individual “request to the insurance company” to cover the costs of therapy (Table 2). A closer look at the latter group revealed that, in the two years (2017 to 2018) before the introduction of the two new reimbursed dHT indications (local tumor compression and painful bone metastasis), more requests for dHT were submitted to insurance companies (15 vs. 9 patients). From 2017 to 2018, dHT was mainly prescribed for the two indications mentioned above (10 of 15) (Supplementary Table S1). With the approval of these two indications, the number of requests to insurance companies decreased, reflecting that an existing clinical demand had been covered. The linear time trend observed over the first two years, with an increase of one patient per semester, could be interpreted as epidemiological growth or may be due to the fact that hyperthermia achieved more visibility within the Swiss (radiation) oncology society. However, the COVID-19 pandemic has clearly influenced case numbers and indications treated from the first semester of 2020 onwards (Figure 2). Due to this confounding bias, a reliable time trend analysis of patient numbers was not possible; however, it is important to note that an uncontrolled increase in case numbers did not happen despite reimbursement of new treatment indications. Taken together, the dHT indications negotiated jointly by the Swiss Federal Office of Public Health and the SHN appear not to have induced a commercially driven increase in patients treated. 

With regard to the referral pattern, our analysis revealed that only 36.8% (35/95) of patients originated in-house and that 63.2% (60/95) patients were referred from external radiation oncology institutions (Figure 3). This shows that a dHT unit in Switzerland, even when integrated into a radiation oncology center, not only treats in-house patients. Patients have been referred for dHT from university hospitals and as well from the proton therapy center at the Paul Scherer Institute explicitly for the treatment of challenging oncological situations (Supplementary Table S2). This indicates that a dHT unit covers an existing demand for specific oncological situations, such as re-irradiation, organ-preserving treatment combinations (bladder and rectal cancer, soft tissue sarcoma) and other complex situations such as inoperable pancreatic cancer, soft tissue sarcoma or bulky, radioresistant tumors. In Switzerland, HT is frequently and incorrectly regarded as a mainly palliative treatment option. In the present analysis, we refute this by showing that 47.4% (45/95) of patients were treated with a curative treatment approach. 

The characteristics of the in-house patients revealed that they generally had a lower performance status and were more likely to be treated with palliative intent. Accordingly, dHT was more often used for the indication “local recurrence and compression”. Patients of low performance status are not fit to travel long distances for dHT, even if they would benefit from a radiosensitizer such as dHT, with its good toxicity profile. For palliative indications, the use of dHT could allow for a reduction in RT dose and thereby improve the tolerability and effect of RT, i.e., regarding pain relief, as has been shown by Chi et al. [39] for painful bone metastases. The referred patients in the present cohort travelled a relatively long mean distance of 62.2 km (SD ± 54.6 km), with a maximum of 238 km, to receive dHT (Supplementary Table S2). This effort is unreasonable for palliative and frail patients, which supports the future higher spatial availability of dHT units in Switzerland. 

The three most commonly reimbursed dHT indications were “local tumor recurrence with compression” (20%), “rectal cancer” (14.7%) and “bladder cancer” (13.7%) (Table 2). Unfortunately, the approval for reimbursement for the most common curative and organ-preserving indications, “rectal cancer” and “bladder cancer”, was withdrawn by July 2021 [20]. Patients treated for the dHT indication “rectal cancer” were mostly referred from external radiotherapy centers (Supplementary Table S2) and predominantly for re-irradiation (71.4%; 10/14 patients, data not shown). More than half (8/14 patients) were treated analogously to the HyRec trial [31] (Supplementary Table S5). The indication “bladder cancer” closes a gap in treatment options for either elderly and frail patients or patients seeking a bladder-sparing treatment approach. Patients were referred from external hospitals for these indications, underlining the demand for this treatment option as well. The SHN board is convinced that there is good evidence for dHT for these two indications [26,27,28,29,32], especially in rectal cancer, since two recent studies showed a promising effect of dHT [30,31]. 

Regarding the other dHT indications, the present analysis revealed that only a few patients are treated for the dHT indication “cervical cancer”, although it is associated with the strongest clinical evidence [21,22,23]. This could be explained by the low incidence of cervical cancer in Switzerland and the fact that this indication only receives direct reimbursement in the case of re-RT and for patients with contraindication to concurrent chemotherapy, which is rarely the case in Switzerland. This is in contrast to, for example, the Netherlands, where dHT is reimbursed in the primary treatment setting in combination with RT and brachytherapy based on evidence from randomized trials [11]. Another observation is the low patient numbers treated for “painful bone metastases”, although its superior effect regarding pain control was shown in a phase III randomized trial [39]. At Kantonsspital Aarau, the combination of dHT+RT for the indication of painful bone metastases was intended to be increasingly used in the future, because, with the longer survival of metastatic patients, long-lasting pain control is also becoming more important. However, because, during the COVID-19 pandemic, non-mandatory treatments were minimized and painful bone metastases could be often sufficiently treated with hypofractionated RT schedules alone, dHT was not offered. After returning to normality in the first semester of 2021, dHT patient numbers almost doubled (Figure 2), reaching the limited capacity of treatment slots for dHT. Therefore, patients with curative treatment indications were prioritized and dHT+RT again was not actively offered to patients qualifying for painful bone metastases. With the increasing dHT treatment capacity and controlled establishment of more dHT units in Switzerland, more patients with painful bone metastases could benefit from the increased analgetic effect of dHT+RT.

The present patterns-of-care analysis was conducted as an inventory/survey of current practice and as the basis for a national objective to define standardized treatment schedules in Switzerland. All reimbursed indications, except for the indications “tumor recurrence and compression” and “painful bone metastasis”, showed relatively standardized treatment schedules in analogy to clinical trials (Supplementary Table S5). In contrast, the indication group “local tumor recurrence with compression” represents a patient collective with enormous heterogeneity regarding primary cancer entities, re-RT status, RT modalities and treatment schedules. The only common denominator is that they were treated mostly with palliative intent (Supplementary Tables S2 and S5). Importantly, these patients often have no other treatment option apart from dHT+RT and local treatment effect has a high impact on their quality of life. Withholding dHT+RT as a last treatment option from these patients would, in our view, be unethical. Because these patients frequently required individually tailored treatment schedules based on their previous treatment, the standardization of the treatment schedules, especially for clinical trials, would also be difficult. It is therefore clear that an analysis of dHT efficacy in this patient group is a challenge. A good example for the standardization of dHT+RT treatment schedules in patients with tumor recurrences is the subgroup of the HyRec trial from Ott et al. [31,52] and the schedule with 5 × 4 Gy once weekly combined with weekly wIRA superficial HT in recurrent breast cancer from Notter et al. [56] for superficial HT. Such innovative study designs and further treatment schedules are required to evaluate and consolidate the effect of dHT in these heterogeneous patient groups.

## 5. Conclusions

To the best of our knowledge, we report the first retrospective analysis of an unselected national patient cohort treated with dHT, evaluating patient numbers over 4.5 years, specific treatment indications, patient characteristics, tumor entities, the referral practice and corresponding treatment schedules in Switzerland.

Nearly 50% of patients were treated with curative intent. Around two thirds of patients were referred from external institutions from all over Switzerland, including from university hospitals and the proton therapy center, for challenging oncologic situations such as re-RT, complex palliative situations, organ-preserving treatment combinations (bladder and rectal cancer, soft tissue sarcoma) and inoperable, bulky or radioresistant tumors. This observation refutes the common prejudice, at least in Switzerland, that HT is only used for palliative situations and clearly underlines the medical need for the combination of dHT+RT.

Patients treated within the reimbursed dHT indications with predominantly curative intent were homogenous subgroups with relatively standardized treatment schedules according to published clinical trials. On the other hand, the present patterns-of-care analysis revealed that patients treated within the two palliative reimbursed indications “tumor local recurrence and compression” and “painful bone metastasis” exhibit immense heterogeneity regarding patient characteristics and treatment schedules, demonstrating the need for standardization as a basis for future clinical studies.

This analysis will provide the basis for standardized national dHT treatment schedules and quality assurance guidelines to consolidate and expand dHT evidence. We think that this insight into dHT practice in Switzerland could be of interest for centers interested in the implementation of a dHT unit and for other HT societies, especially regarding reimbursement policy, and could also foster international study collaborations.

## Figures and Tables

**Table 1:** Indications for deep hyperthermia (dHT) with granted reimbursement in Switzerland [18,19,20] are stated with specifications and underlying evidence.

Deep HT Indication	Specification	Reimbursement Status per Time Period			Evidence
		2017 2018	2019 2020	2021 2022	
**Cervical cancer**	- Prior irradiation - Contraindication for ChT				[12,21,22,23]
**Bladder cancer**	- Function preservation - Prior irradiation - Contraindication for ChT				[24,25,26,27,28,29]
**Rectal cancer**	- Function preservation - Local recurrence in pre-irradiated area - Contraindication for ChT				[27,30,31,32]
**Soft tissue sarcoma**	- Function preservation - Contraindication for ChT				[33,34,35]
**Pancreatic cancer**	- Locally advanced, initially inoperable tumor				[36,37,38]
**Local tumor recurrence with compression**	- Patients with local tumor recurrence and symptoms due to tumor compression (palliative situation) - Tumor depth > 5 cm				[2]
**Painful bone metastasis**	- Located in the pelvis or vertebral bodies - Tumor depth > 5 cm				[39]

Prerequisites are (i) combination with radiotherapy (RT), (ii) the indication has to be presented and confirmed at the Swiss Hyperthermia Network (SHN) tumor board, (iii) the combined dHT + RT has to be performed at an institution affiliated with the SHN. The reimbursement status is indicated per time period and coded with underlying colors. Green = time-unrestricted reimbursement; yellow = reimbursed indications limited for two further years; red = indications no longer reimbursed; grey = initially not reimbursed indications (request for insurance cover was required). Abbreviations: ChT: chemotherapy, HT: hyperthermia.

**Table 2:** Patient and tumor characteristics with treatment indications, referral status and deep hyperthermia treatment adherence. Specifications of “reimbursed dHT indications” are given in Table 1.

Patient Characteristics	
	Total (*n* = 95)
**Sex**	
Male	55 (57.9%)
Female	40 (42.1%)
**Age**	
Mean (SD)	63.1 (14.2)
Median [Min, Max]	65 [18, 88]
**ECOG**	
0	47 (49.5%)
1	39 (41.1%)
2	9 (9.5%)
**Reimbursed dHT indications**	
Cervical cancer	2 (2.1%)
Bladder cancer	13 (13.7%)
Rectal cancer	14 (14.7%)
Soft tissue sarcoma	8 (8.4%)
Pancreatic cancer	8 (8.4%)
Local tumor recurrence with compression	19 (20.0%)
Painful bone metastasis	7 (7.4%)
Request for insurance cover	24 (25.3%)
**Primary cancer entities**	
Cervical cancer	3 (3.2%)
Bladder cancer	15 (15.8%)
Rectal cancer	21 (22.1%)
Soft tissue sarcoma	13 (13.7%)
Pancreatic cancer	8 (8.4%)
Prostate cancer	7 (7.4%)
Anal cancer	4 (4.2%)
Colon cancer	6 (6.3%)
Others	18 (18.9%)
**Treatment intention**	
Curative	45 (47.4%)
Palliative	50 (52.6%)
**Re-irradiation**	
No	55 (57.9%)
Yes	40 (42.1%)
**Treatment within a study protocol**	
No	66 (69.5%)
Yes	7 (7.4%)
Analogous to protocol	22 (23.2%)
**Patient origin**	
In-house patient	35 (36.8%)
Referred from external hospital	60 (63.2%)
**Patient origin (specified)**	
Intra-cantonal	26 (43.3%)
Extra-cantonal	34 (56.7%)
**Distance to referring hospital (km)**	
Median [Min, Max]	42 [23, 238]
Mean (SD)	61.5 (54.3)
**Place of treatment**	
RT at referring institution, dHT at KSA	40 (42.1%)
dHT+RT at KSA	49 (51.6%)
HT and only RT at the same day at KSA, remaining RT at referring institution	6 (6.3%)
**All prescribed dHT sessions received**	
No	6 (6.3%)
Yes	89 (93.7%)

Abbreviations: dHT: deep hyperthermia, dHT+RT: combined dHT and RT, ECOG: Eastern Cooperative Oncology Group, intra and extra-cantonal: cantons in Switzerland are equivalent to states, provinces or regions in other countries, KSA: Kantonsspital Aarau (=dHT center), Others: the definition is given in the text, RT: radiotherapy, SD: standard deviation.

**Table 3:** Treatment characteristics for specific patient subgroups comparing in-house vs. referred patients, primary RT vs. re-RT and curative vs. palliative intention.

Treatment Characteristics by							
	Referral Status		Re-Irradiation Status		Treatment Intention		
	In-House Patients	Referred from External Hospital	No	Yes	Curative	Palliative	Total
	(*n* = 35)	(*n* = 59)	(*n* = 55)	(*n* = 39)	(*n* = 45)	(*n* = 49)	(*n* = 94)
**HT frequency**							
Once per week	14 (40.0%)	35 (59.3%)	36 (65.5%)	13 (33.3%)	32 (71.1%)	17 (34.7%)	49 (52.1%)
Once to twice per week	21 (60.0%)	24 (40.7%)	19 (34.5%)	26 (66.7%)	13 (28.9%)	32 (65.3%)	45 (47.9%)
**No. of dHT sessions**							
Mean (SD)	5.17 (1.44)	5.29 (2.20)	5.53 (1.91)	4.85 (1.94)	5.60 (1.99)	4.92 (1.86)	5.24 (1.94)
Median [Min, Max]	5 [1, 8]	5 [1, 10]	6 [1, 10]	5 [1, 8]	6 [1, 10]	5 [1, 10]	5 [1, 10]
**Total no. of RT fractions**							
Mean (SD)	20.1 (8.11)	22.6 (9.27)	24.9 (6.42)	17.1 (9.90)	26.7 (5.98)	17.0 (8.63)	21.7 (8.89)
Median [Min, Max]	23 [4, 35]	25 [4, 38]	27 [10, 35]	15 [4, 38]	28 [4, 38]	15 [4, 35]	25 [4, 38]
**Dose/fraction (Gy)**							
Mean (SD)	2.46 (1.04)	2.51 (1.51)	2.14 (0.413)	3.00 (1.94)	2.14 (0.950)	2.82 (1.57)	2.49 (1.35)
Median [Min, Max]	2 [1.8, 7.5]	2 [1.8, 9]	2 [1.8, 3]	2.5 [1.8, 9]	2 [1.8, 8]	2.5 [1.8, 9]	2 [1.8, 9]
**Boost included**							
No	27 (77.1%)	48 (81.4%)	38 (69.1%)	37 (94.9%)	29 (64.4%)	46 (93.9%)	75 (79.8%)
Yes	8 (22.9%)	11 (18.6%)	17 (30.9%)	2 (5.1%)	16 (35.6%)	3 (6.1%)	19 (20.2%)
**Total dose (Gy)**							
Mean (SD)	43.6 (10.6)	47.6 (13.8)	51.1 (8.85)	39.2 (14.3)	53.3 (8.55)	39.6 (12.7)	46.2 (12.8)
Median [Min, Max]	45 [24, 70]	50 [12.5, 76]	50.4 [30, 71]	32 [12.5, 76]	50.4 [32, 76]	36 [12.5, 71]	50 [12.5, 76]
**RT interval**							
1×/week	0 (0%)	1 (1.7%)	0 (0%)	1 (2.6%)	0 (0%)	1 (2.0%)	1 (1.1%)
2×/week	2 (5.7%)	4 (6.8%)	0 (0%)	6 (15.4%)	1 (2.2%)	5 (10.2%)	6 (6.4%)
3×/week	0 (0%)	0 (0%)	0 (0%)	0 (0%)	0 (0%)	0 (0%)	0 (0%)
4×/week	4 (11.4%)	5 (8.5%)	7 (12.7%)	2 (5.1%)	4 (8.9%)	5 (10.2%)	9 (9.6%)
5×/week	29 (82.9%)	49 (83.1%)	48 (87.3%)	30 (76.9%)	40 (88.9%)	38 (77.6%)	78 (83.0%)
**RT modality**							
EBRT	33 (94.3%)	46 (78.0%)	50 (90.9%)	29 (74.4%)	38 (84.4%)	41 (83.7%)	79 (84.0%)
HDR—brachytherapy	0 (0%)	4 (6.8%)	0 (0%)	4 (10.3%)	1 (2.2%)	3 (6.1%)	4 (4.3%)
Protons	0 (0%)	9 (15.3%)	5 (9.1%)	4 (10.3%)	6 (13.3%)	3 (6.1%)	9 (9.6%)
SBRT	2 (5.7%)	0 (0%)	0 (0%)	2 (5.1%)	0 (0%)	2 (4.1%)	2 (2.1%)

One patient stopped treatment very early and was excluded from the treatment characteristics table. Abbreviations: dHT: deep hyperthermia, EBRT: external body radiotherapy, Gy: Gray, HDR: high dose rate, RT: radiotherapy, SBRT: stereotactic body radiotherapy, SD: standard deviation.

## Data Availability

The data presented in this study are available on request from the corresponding author. The data are not publicly available due to privacy and ethical reasons.

## Supplementary Materials

The following are available online at https://www.mdpi.com/article/10.3390/cancers14051175/s1, Figure S1: Number of cases presented at the Swiss Hyperthermia Network tumor board by primary cancer entity. Table S1: Patients and tumor characteristics of patients treated with deep hyperthermia who required an individual request for insurance cover. Table S2: Patient characteristics regarding referral status, re-irradiation status and by treatment indication. Table S3: Patient, tumor and treatment characteristics according to treatment protocol. Table S4: Patient characteristics by gender. Table S5: Deep hyperthermia and combined radiotherapy treatment schedules by specific reimbursed dHT indications.
