# Supplementary material for: Present Practice of Radiative Deep Hyperthermia in Combination with Radiotherapy in Switzerland
Source: Cancers (Basel). 2022 Feb 24;14(5):1175. doi: 10.3390/cancers14051175 (PMC8909523; doi:10.3390/cancers14051175)
Supplement: Supplementary file 1 [file cancers-14-01175-s001.zip › cancers-1595118-supplementary.pdf]

cancers MDPI

Supplementary Material

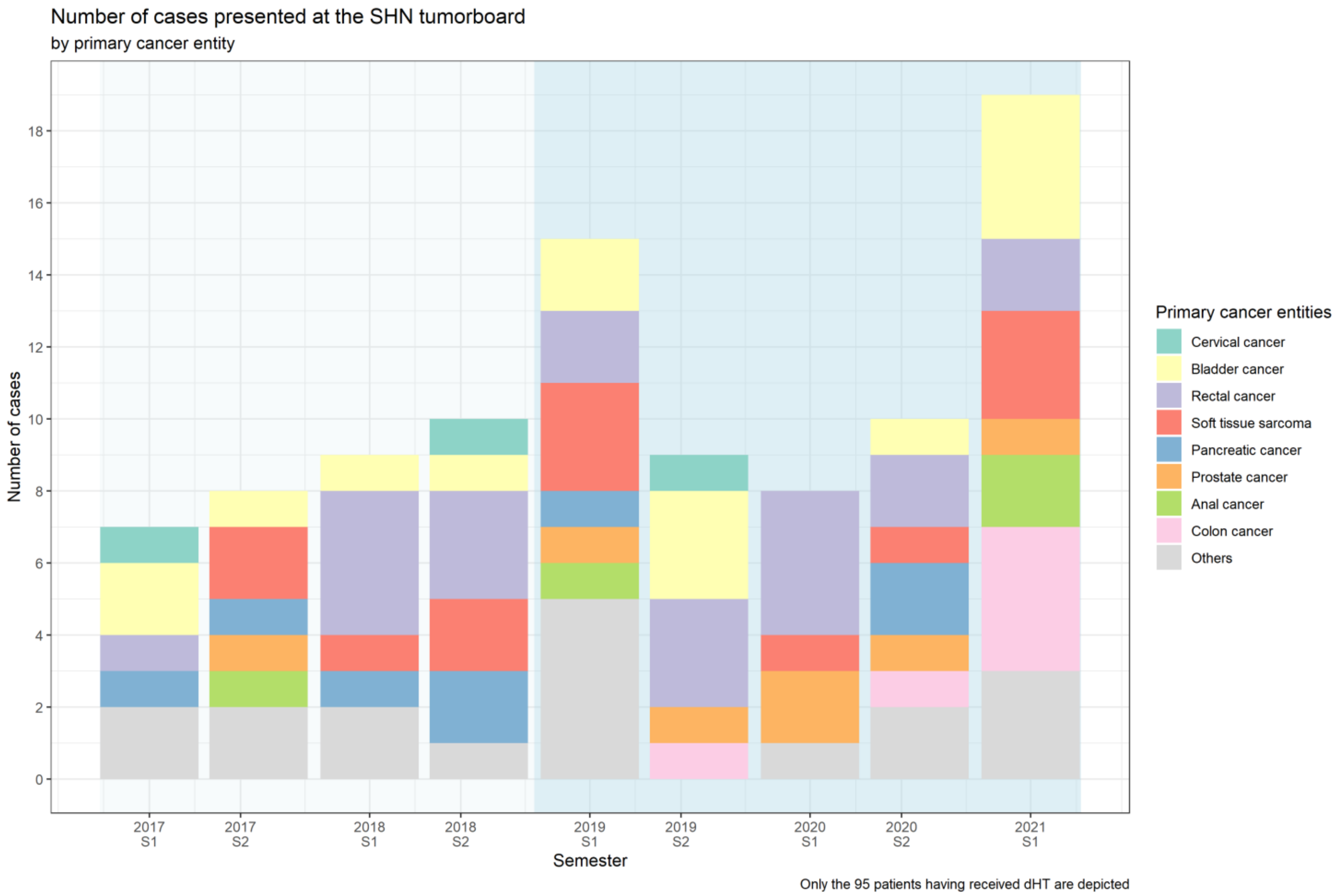

Figure S1. Time trend of patients treated with combined deep hyperthermia (dHT) and radiotherapy. Bar chart with patient numbers receiving dHT in the time period from January, 2017 to June, 2021 are depicted per semester (S1 and S2) and basic tumor entity. Two new reimbursed indications were granted as from 2019 (blue shaded background). COVID-19 lockdown in Switzerland was during 1st semester 2020 (11 March to 26 April 2020).

Table S1. Patient and tumor characteristics of 24 of 95 patients treated from January, 2017 to June, 2021 with deep hyperthermia and radiotherapy (dHT+RT), who required an individual request for insurance cover. The respective indication for dHT and the reasons why reimbursement was requested are stated. Ten patients (8+2, gray shaded background) were treated from 2017 to 2018 for tumor local recurrence or painful bone metastasis, respectively. During this time period these indications required a request for insurance cover, as they have been only a reimbursed indication since 2019. The other five patients treated with dHT+RT from 2017 to 2018 and the nine patients treated since 2019 arere depicted below.

No. (total 24)	Time Period	Primary Tumor Entity	Indication for Deep Hyperthermia	Treatment Intent	Re-RT	Reason for "Request for Insurance Cover"
8	2017-2018	Mixed	Tumor recurrence and compression	mixed	mixed	Treated 2017-2018. Indication has been only reimbursed since 2019
2	2017-2018	Mixed	Painful bone metastasis	mixed	mixed	Treated 2017-2018. Indication has been only reimbursed since 2019
1	2017-2018	Desmoid	Recurrent tumor after several treatments	Curative	No	Not a classical sarcoma
1	2017-2018	Chordoma [54]	Bulky, radioresistant tumor	Curative	No	Not a classical sarcoma
1	2017-2018	Ewing Sarcoma	Tumorbulk abutting the spinal cord	Curative	No	Not a classical sarcoma. No local recurrence, no palliative situation
1	2017-2018	Endometrial cancer	Local recurrence in pre-irradiated area	Curative	Yes	Recurrence, but no compression and in a curative setting
1	2017-2018	CUP	Recurrence in pre-irradiated area	Curative	Yes	Treated with curative intent
1	Since 2019	NSCLC	Painful bone metastasis in tibia	Palliative	No	Location was not spine or pelvis
1	Since 2019	Prostate cancer	LN recurrence, Pre-irradiated	Palliative	Yes	Small LN, and not yet a compression
1	Since 2019	Colon cancer	Bulky metastasis with compression of aorta	Palliative	No	No recurrence, primary tumor bulk
1	Since 2019	Urothelial cancer of ureter	Bulky disease with compression, dose-limiting area	Curative	No	No recurrence, primary tumor bulk, curative intention
1	Since 2019	Rectal and prostate cancer	Bulky disease with compression in the pelvis	Curative	No	Double carcinoma
1	Since 2019	Prostate cancer (sarcomatoid)	Bulky disease with compression in the pelvis	Curative	Yes	No recurrence and in curative intention
1	Since 2019	Prostate cancer	Recurrence in the pre-irradiated area	Curative	Yes	Treated with curative intention
2	Since 2019	Anal cancer	Clinical study [49]	Curative	No	No present indication

Abbreviations: CUP: Cancer of unknown primary, LN: Lymph node, NSCLC: Non-small cell lung cancer, Re-RT: Re-irradiation.

Table S2. Patient characteristics regarding referral status (column 1-2), re-irradiation status (column 3-4) and by treatment indication (column 5-6). Total numbers are stated in column 7. Due to low case numbers, high volume of multiple testing and too many associated factors, statistical analyses regarding significance of differences would have been statistically inadequate and were therefore omitted.

Patients Characteristics by							
	**Referral status**		**Re-Irradiation Status**		**Treatment Intention**		
	**In-house patients**	**Referred from external hospital**	**No**	**Yes**	**Curative**	**Palliative**	**Total**
	**(n = 35)**	**(n = 60)**	**(n = 55)**	**(n = 40)**	**(n = 45)**	**(n = 50)**	**(n = 95)**
**Sex**							
Male	15 (42.9%)	40 (66.7%)	34 (61.8%)	21 (52.5%)	29 (64.4%)	26 (52.0%)	55 (57.9%)
Female	20 (57.1%)	20 (33.3%)	21 (38.2%)	19 (47.5%)	16 (35.6%)	24 (48.0%)	40 (42.1%)
**Age**							
Mean (SD)	66.4 (12.7)	61.1 (14.7)	63.1 (14.1)	63.1 (14.4)	61.8 (15.3)	64.2 (13.1)	63.1 (14.2)
Median [Min, Max]	63 [35, 88]	65 [18, 81]	63 [18, 85]	65 [24, 88]	65 [18, 85]	65 [24, 88]	65 [18, 88]
**ECOG**							
0	13 (37.1%)	34 (56.7%)	38 (69.1%)	9 (22.5%)	34 (75.6%)	13 (26.0%)	47 (49.5%)
1	16 (45.7%)	23 (38.3%)	13 (23.6%)	26 (65.0%)	10 (22.2%)	29 (58.0%)	39 (41.1%)
2	6 (17.1%)	3 (5.0%)	4 (7.3%)	5 (12.5%)	1 (2.2%)	8 (16.0%)	9 (9.5%)
**Reimbursed dHT indications**							
Cervical cancer	1 (2.9%)	1 (1.7%)	1 (1.8%)	1 (2.5%)	2 (4.4%)	0 (0%)	2 (2.1%)
Bladder cancer	4 (11.4%)	9 (15.0%)	13 (23.6%)	0 (0%)	12 (26.7%)	1 (2.0%)	13 (13.7%)
Rectal cancer	3 (8.6%)	11 (18.3%)	4 (7.3%)	10 (25.0%)	7 (15.6%)	7 (14.0%)	14 (14.7%)
Soft tissue sarcoma	2 (5.7%)	6 (10.0%)	6 (10.9%)	2 (5.0%)	7 (15.6%)	1 (2.0%)	8 (8.4%)
Pancreatic cancer	2 (5.7%)	6 (10.0%)	8 (14.5%)	0 (0%)	7 (15.6%)	1 (2.0%)	8 (8.4%)
Local tumor recurrence with compression	10 (28.6%)	9 (15.0%)	8 (14.5%)	11 (27.5%)	0 (0%)	19 (38.0%)	19 (20.0%)
Painful bone metastasis	1 (2.9%)	6 (10.0%)	3 (5.5%)	4 (10.0%)	0 (0%)	7 (14.0%)	7 (7.4%)

Request for insurance cover	12 (34.3%)	12 (20.0%)	12 (21.8%)	12 (30.0%)	10 (22.2%)	14 (28.0%)	24 (25.3%)
**Primary cancer entities**							
Cervical cancer	2 (5.7%)	1 (1.7%)	1 (1.8%)	2 (5.0%)	2 (4.4%)	1 (2.0%)	3 (3.2%)
Bladder cancer	6 (17.1%)	9 (15.0%)	14 (25.5%)	1 (2.5%)	12 (26.7%)	3 (6.0%)	15 (15.8%)
Rectal cancer	7 (20.0%)	14 (23.3%)	6 (10.9%)	15 (37.5%)	7 (15.6%)	14 (28.0%)	21 (22.1%)
Soft tissue sarcoma	2 (5.7%)	11 (18.3%)	7 (12.7%)	6 (15.0%)	8 (17.8%)	5 (10.0%)	13 (13.7%)
Pancreatic cancer	2 (5.7%)	6 (10.0%)	8 (14.5%)	0 (0%)	7 (15.6%)	1 (2.0%)	8 (8.4%)
Prostate cancer	5 (14.3%)	2 (3.3%)	3 (5.5%)	4 (10.0%)	1 (2.2%)	6 (12.0%)	7 (7.4%)
Anal cancer	1 (2.9%)	3 (5.0%)	2 (3.6%)	2 (5.0%)	2 (4.4%)	2 (4.0%)	4 (4.2%)
Colon cancer	1 (2.9%)	5 (8.3%)	4 (7.3%)	2 (5.0%)	1 (2.2%)	5 (10.0%)	6 (6.3%)
Others	9 (25.7%)	9 (15.0%)	10 (18.2%)	8 (20.0%)	5 (11.1%)	13 (26.0%)	18 (18.9%)
**Treatment intention**							
Curative	15 (42.9%)	30 (50.0%)	37 (67.3%)	8 (20.0%)	---	---	45 (47.4%)
Palliative	20 (57.1%)	30 (50.0%)	18 (32.7%)	32 (80.0%)	---	---	50 (52.6%)
**Re-irradiation**							
No	22 (62.9%)	33 (55.0%)	---	---	37 (82.2%)	18 (36.0%)	55 (57.9%)
Yes	13 (37.1%)	27 (45.0%)	---	---	8 (17.8%)	32 (64.0%)	40 (42.1%)
**Treatment within a study protocol**							
No	28 (80.0%)	38 (63.3%)	32 (58.2%)	34 (85.0%)	20 (44.4%)	46 (92.0%)	66 (69.5%)
Yes	1 (2.9%)	6 (10.0%)	7 (12.7%)	0 (0%)	7 (15.6%)	0 (0%)	7 (7.4%)
Analogous to protocol	6 (17.1%)	16 (26.7%)	16 (29.1%)	6 (15.0%)	18 (40.0%)	4 (8.0%)	22 (23.2%)
**Patient origin**							
In-house patient	---	---	22 (40.0%)	13 (32.5%)	15 (33.3%)	20 (40.0%)	35 (36.8%)
Referred from external hospital	---	---	33 (60.0%)	27 (67.5%)	30 (66.7%)	30 (60.0%)	60 (63.2%)
**Patient origin (specified)**							
Intra-cantonal	---	25 (42.4%)	19 (57.6%)	6 (23.1%)	16 (53.3%)	9 (31.0%)	25 (42.4%)
Extra-cantonal	---	34 (57.6%)	14 (42.4%)	20 (76.9%)	14 (46.7%)	20 (69.0%)	34 (57.6%)

**Distance to referring hospital (km)**							
Median [Min, Max]	---	42 [23, 238]	27 [23, 238]	48 [23, 238]	30 [23, 238]	48 [23, 238]	42 [23, 238]
Mean (SD)	---	61.5 (54.3)	48.6 (47.7)	76.0 (58.3)	49.4 (46.1)	72.5 (59.4)	61.5 (54.3)
**Place of treatment**							
RT at referring institution, dHT at KSA	0 (0%)	40 (66.7%)	23 (41.8%)	17 (42.5%)	20 (44.4%)	20 (40.0%)	40 (42.1%)
dHT+RT at KSA	35 (100%)	14 (23.3%)	30 (54.5%)	19 (47.5%)	22 (48.9%)	27 (54.0%)	49 (51.6%)
HT and only RT at the same day at KSA, remaining RT at referring institution	0 (0%)	6 (10.0%)	2 (3.6%)	4 (10.0%)	3 (6.7%)	3 (6.0%)	6 (6.3%)
**All prescribed dHT sessions received**							
No	1 (2.9%)	5 (8.3%)	5 (9.1%)	1 (2.5%)	4 (8.9%)	2 (4.0%)	6 (6.3%)
Yes	34 (97.1%)	55 (91.7%)	50 (90.9%)	39 (97.5%)	41 (91.1%)	48 (96.0%)	89 (93.7%)

Abbreviations: ECOG: Eastern Cooperative Oncology Group, dHT: Deep hyperthermia, dHT+RT: deep hyperthermia with radiotherapy, SD: standard deviation, Tx: treatment, RT: radiotherapy, KSA: Kantonsspital Aarau.

Table S3. Patient (A) and treatment characteristics (B) are depicted according to treatment protocol. "No" means that the patient was treated in routine clinical practice. "Yes" means that the patient was treated within a clinical trial. A patient treated "analogous to a protocol" means that (1) he could not have been included in the protocol due to contraindications but was treated according to protocol, or (2) that this protocol had not been opened at the Kantonsspital Aarau, or (3) that the protocol had already closed to recruitment, or (4) the study treatment schedule had to be adapted for an individualized approach. Due to low case numbers, high volume of multiple testing and too many associated factors, statistical analyses regarding significance of differences would have been statistically inadequate and were therefore omitted.

**S3 A**	**Patient Characteristics by Protocol Status**			
	**No**	**Yes**	**Analogous to Protocol**	**Total**
	**(n = 66)**	**(n = 7)**	**(n = 22)**	**(n = 95)**
**Sex**				
Male	35 (53.0%)	6 (85.7%)	14 (63.6%)	55 (57.9%)
Female	31 (47.0%)	1 (14.3%)	8 (36.4%)	40 (42.1%)
**Age**				
Mean (SD)	64.3 (14.3)	66.0 (7.55)	58.6 (14.9)	63.1 (14.2)
Median [Min, Max]	68 [24, 88]	64 [57, 80]	60 [18, 85]	65 [18, 88]
**ECOG**				
0	26 (39.4%)	6 (85.7%)	15 (68.2%)	47 (49.5%)
1	32 (48.5%)	1 (14.3%)	6 (27.3%)	39 (41.1%)
2	8 (12.1%)	0 (0%)	1 (4.5%)	9 (9.5%)
**Reimbursed dHT indications**				
Cervical cancer	2 (3.0%)	0 (0%)	0 (0%)	2 (2.1%)
Bladder cancer	6 (9.1%)	3 (42.9%)	4 (18.2%)	13 (13.7%)
Rectal cancer	6 (9.1%)	0 (0%)	8 (36.4%)	14 (14.7%)
Soft tissue sarcoma	7 (10.6%)	0 (0%)	1 (4.5%)	8 (8.4%)
Pancreatic cancer	0 (0%)	2 (28.6%)	6 (27.3%)	8 (8.4%)
Local tumor recurrence with compression	19 (28.8%)	0 (0%)	0 (0%)	19 (20.0%)
Painful bone metastasis	7 (10.6%)	0 (0%)	0 (0%)	7 (7.4%)
Request for insurance cover	19 (28.8%)	2 (28.6%)	3 (13.6%)	24 (25.3%)
**Primary cancer entities**				
Cervical cancer	3 (4.5%)	0 (0%)	0 (0%)	3 (3.2%)
Bladder cancer	8 (12.1%)	3 (42.9%)	4 (18.2%)	15 (15.8%)
Rectal cancer	13 (19.7%)	0 (0%)	8 (36.4%)	21 (22.1%)
Soft tissue sarcoma	12 (18.2%)	0 (0%)	1 (4.5%)	13 (13.7%)
Pancreatic cancer	0 (0%)	2 (28.6%)	6 (27.3%)	8 (8.4%)
Prostate cancer	7 (10.6%)	0 (0%)	0 (0%)	7 (7.4%)
Anal cancer	2 (3.0%)	2 (28.6%)	0 (0%)	4 (4.2%)
Colon cancer	5 (7.6%)	0 (0%)	1 (4.5%)	6 (6.3%)

Others	16 (24.2%)	0 (0%)	2 (9.1%)	18 (18.9%)
**Treatment intention**				
Curative	20 (30.3%)	7 (100%)	18 (81.8%)	45 (47.4%)
Palliative	46 (69.7%)	0 (0%)	4 (18.2%)	50 (52.6%)
**Re-irradiation**				
No	32 (48.5%)	7 (100%)	16 (72.7%)	55 (57.9%)
Yes	34 (51.5%)	0 (0%)	6 (27.3%)	40 (42.1%)
**Patient origin**				
In-house patient	28 (42.4%)	1 (14.3%)	6 (27.3%)	35 (36.8%)
Referred from external hospital	38 (57.6%)	6 (85.7%)	16 (72.7%)	60 (63.2%)
**Patient origin (specified)**				
Intra-cantonal	13 (35.1%)	3 (50.0%)	9 (56.3%)	25 (42.4%)
Extra-cantonal	24 (64.9%)	3 (50.0%)	7 (43.8%)	34 (57.6%)
**Distance to referring hospital (km)**				
Median [Min, Max]	48 [23, 238]	33 [23, 59]	30 [23, 116]	42 [23, 238]
Mean (SD)	71.5 (61.4)	36.8 (17.3)	43.0 (31.1)	61.5 (54.3)
**Place of treatment**				
RT at referring institution, dHT at KSA	25 (37.9%)	2 (28.6%)	13 (59.1%)	40 (42.1%)
dHT+RT at KSA	37 (56.1%)	5 (71.4%)	7 (31.8%)	49 (51.6%)
HT and only RT at the same day at KSA, remaining RT at referring hospital	4 (6.1%)	0 (0%)	2 (9.1%)	6 (6.3%)
**All prescribed dHT sessions received**				
No	4 (6.1%)	2 (28.6%)	0 (0%)	6 (6.3%)
Yes	62 (93.9%)	5 (71.4%)	22 (100%)	89 (93.7%)

S3 B	Treatment Characteristics by Protocol Status			
	**No**	**Yes**	**Analogous to Protocol**	**Total**
	**(n = 65)**	**(n = 7)**	**(n = 22)**	**(n = 94)**
**HT frequency**				
Once per week	30 (46.2%)	5 (71.4%)	14 (63.6%)	49 (52.1%)
Once to twice per week	35 (53.8%)	2 (28.6%)	8 (36.4%)	45 (47.9%)
**Nos. of dHT sessions**				
Mean (SD)	4.94 (1.99)	4.71 (2.14)	6.32 (1.32)	5.24 (1.94)
Median [Min, Max]	5 [1, 10]	6 [1, 7]	6 [4, 10]	5 [1, 10]
**Total nos. of RT fractions**				
Mean (SD)	18.7 (8.92)	29.7 (1.60)	27.9 (4.07)	21.7 (8.89)
Median [Min, Max]	16 [4, 35]	31 [28, 31]	28 [20, 38]	25 [4, 38]
**Dose/fraction (Gy)**				
Mean (SD)	2.75 (1.55)	1.86 (0.0976)	1.93 (0.207)	2.49 (1.35)
Median [Min, Max]	2.15 [1.8, 9]	1.80 [1.8, 2]	1.80 [1.8, 2.5]	2.00 [1.8, 9]
**Boost included**				
No	56 (86.2%)	3 (42.9%)	16 (72.7%)	75 (79.8%)
Yes	9 (13.8%)	4 (57.1%)	6 (27.3%)	19 (20.2%)
**Total dose (Gy)**				
Mean (SD)	42.6 (13.0)	55.4 (2.38)	53.7 (9.09)	46.2 (12.8)
Median [Min, Max]	45.0 [12.5, 71]	55.8 [50.4, 58.2]	54.9 [36, 76]	50.0 [12.5, 76]
**RT interval**				
1x/week	1 (1.5%)	0 (0%)	0 (0%)	1 (1.1%)
2x/week	6 (9.2%)	0 (0%)	0 (0%)	6 (6.4%)
3x/week	0 (0%)	0 (0%)	0 (0%)	0 (0%)
4x/week	9 (13.8%)	0 (0%)	0 (0%)	9 (9.6%)
5x/week	49 (75.4%)	7 (100%)	22 (100%)	78 (83.0%)
**RT modality**				
EBRT	53 (81.5%)	7 (100%)	19 (86.4%)	79 (84.0%)
HDR - brachytherapy	4 (6.2%)	0 (0%)	0 (0%)	4 (4.3%)
Protons	6 (9.2%)	0 (0%)	3 (13.6%)	9 (9.6%)
SBRT	2 (3.1%)	0 (0%)	0 (0%)	2 (2.1%)

One patient stopped treatment very early and was excluded from the treatment characteristics table.

Abbreviations: EBRT: external body radiotherapy, ECOG: Eastern Cooperative Oncology Group, fx: fraction, Gy: Gray, HDR: high dose rate, HT: hyperthermia, KSA: Kantonsspital Aarau (dHT center), RT: radiotherapy, SBRT: stereotactic body radiotherapy, SD: standard deviation, Tx: treatment.

Table S4. Patient characteristics are depicted by gender. Due to low case numbers, high volume of multiple testing and too many associated factors, statistical analyses regarding significance of differences would have been statistically inadequate and were therefore omitted.

Patient Characteristics by Gender			
	**Male**	**Female**	**Total**
	**(n = 55)**	**(n = 40)**	**(n = 95)**
**Age**			
Mean (SD)	66.2 (13.3)	58.8 (14.3)	63.1 (14.2)
Median [Min, Max]	68 [18, 88]	61 [24, 85]	65 [18, 88]
**ECOG**			
0	30 (54.5%)	17 (42.5%)	47 (49.5%)
1	22 (40.0%)	17 (42.5%)	39 (41.1%)
2	3 (5.5%)	6 (15.0%)	9 (9.5%)
**Reimbursed dHT indications**			
Cervical cancer	0 (0%)	2 (5.0%)	2 (2.1%)
Bladder cancer	11 (20.0%)	2 (5.0%)	13 (13.7%)
Rectal cancer	8 (14.5%)	6 (15.0%)	14 (14.7%)
Soft tissue sarcoma	4 (7.3%)	4 (10.0%)	8 (8.4%)
Pancreatic cancer	5 (9.1%)	3 (7.5%)	8 (8.4%)
Local tumor recurrence with compression	7 (12.7%)	12 (30.0%)	19 (20.0%)
Painful bone metastasis	6 (10.9%)	1 (2.5%)	7 (7.4%)
Request for insurance cover	14 (25.5%)	10 (25.0%)	24 (25.3%)
**Primary cancer entities**			
Cervical cancer	0 (0%)	3 (7.5%)	3 (3.2%)
Bladder cancer	11 (20.0%)	4 (10.0%)	15 (15.8%)
Rectal cancer	14 (25.5%)	7 (17.5%)	21 (22.1%)
Soft tissue sarcoma	8 (14.5%)	5 (12.5%)	13 (13.7%)
Pancreatic cancer	5 (9.1%)	3 (7.5%)	8 (8.4%)
Prostate cancer	7 (12.7%)	0 (0%)	7 (7.4%)
Anal cancer	1 (1.8%)	3 (7.5%)	4 (4.2%)
Colon cancer	1 (1.8%)	5 (12.5%)	6 (6.3%)
Others	8 (14.5%)	10 (25.0%)	18 (18.9%)
**Treatment intention**			
Curative	29 (52.7%)	16 (40.0%)	45 (47.4%)
Palliative	26 (47.3%)	24 (60.0%)	50 (52.6%)
**Re-irradiation**			
No	34 (61.8%)	21 (52.5%)	55 (57.9%)
Yes	21 (38.2%)	19 (47.5%)	40 (42.1%)

**Treatment within a study protocol**			
No	35 (63.6%)	31 (77.5%)	66 (69.5%)
Yes	6 (10.9%)	1 (2.5%)	7 (7.4%)
Analogous to protocol	14 (25.5%)	8 (20.0%)	22 (23.2%)
**Patient origin**			
In-house patient	15 (27.3%)	20 (50.0%)	35 (36.8%)
Referred from external hospital	40 (72.7%)	20 (50.0%)	60 (63.2%)
**Patient origin (specified)**			
Intra-cantonal	18 (45.0%)	7 (36.8%)	25 (42.4%)
Extra-cantonal	22 (55.0%)	12 (63.2%)	34 (57.6%)
**Distance to referring hospital (km)**			
Median [Min, Max]	42 [23, 238]	48 [23, 238]	42 [23, 238]
Mean (SD)	59.4 (52.8)	65.7 (58.5)	61.5 (54.3)
**Place of treatment**			
RT at referring institution, dHT at KSA	22 (40.0%)	18 (45.0%)	40 (42.1%)
dHT+RT at KSA	27 (49.1%)	22 (55.0%)	49 (51.6%)
HT and only RT at the same day at KSA, remaining RT at referring institution	6 (10.9%)	0 (0%)	6 (6.3%)
**All prescribed dHT sessions received**			
No	4 (7.3%)	2 (5.0%)	6 (6.3%)
Yes	51 (92.7%)	38 (95.0%)	89 (93.7%)

Abbreviations: dHT: deep hyperthermia, dHT+RT: combined dHT and RT, ECOG: Eastern Cooperative Oncology Group, Intra and extra-cantonal: cantons in Switzerland are equivalent to states, provinces or regions in other countries, KSA: Kantonsspital Aarau (= dHT center), Others: the definition is given in the text, RT: radiotherapy, SD: standard deviation.

Table S5. Summary of applied deep hyperthermia and combined radiotherapy (dHT+RT) treatment schedules of the 71 patients treated by specific reimbursed dHT indication. One patient stopped dHT+RT after three RT fractions due to reasons unrelated to treatment and was therefore excluded from the treatment schedule analysis. As far as possible, groups of similar schedules were formed and numbers of respective patients noted in the 1st column. If to many different schedules existed, they were taken together and denoted with patient numbers and ″(mix)″, giving ranges with maximum and minimum. If available, the corresponding study was referred.

Cervical cancer

Nos.	Fx	Dose/fx (Gy)	Total dose (Gy)	Included boost	RT**frequency	RT modality	dHT sessions	dHT frequency**	Treatment intent	Re-RT	Ref
1x	25	1.8 Gy	45 Gy		5	EBRT	5	1x	curativ	yes	
1x	28	1.8 Gy	50.4 Gy	Brachy	5	EBRT	5	1x	curativ	no	[12]

Bladder cancer

Nos.	Fx	Dose/fx (Gy)	Total dose (Gy)	Included boost	RT**frequency	RT modality	dHT sessions	dHT frequency**	Treatment intent	Re-RT	Ref
3x	16	3 Gy	48 Gy	4 × 3 Gy = 12 Gy	4	EBRT	4	1x	curativ	no	[26]
2x	20	2.5 Gy	50 Gy		5	EBRT	2*, 5	1x	curativ	no	[26]
4x	31	1.8 Gy	55.8 Gy	6 × 1.8 Gy = 10.8 Gy	5	EBRT	1*, 5-7	1x	curativ	no	[51]
2x	31	1.8 Gy	58.2 Gy	6 × 2.2 Gy = 13.2 Gy	5	EBRT	3*, 6	1x	curativ	no	[51]
2x(mix)	16-33	1.8-2.5 Gy	40-59.4 Gy	yes	4-5	EBRT	4-8	1-2x	cur/pall	no	

Rectal cancer

Nos.	Fx	Dose/fx (Gy)	Total dose (Gy)	Included boost	RT**frequency	RT modality	dHT sessions	dHT frequency**	Treatment intent	Re-RT	Ref
6x	25	1.8 Gy	45 Gy		5	EBRT	5-8	1-2x	cur/pall	yes/no	[31,52]
2x	28	1.8 Gy	50.4 Gy	3 × 1.8 Gy = 5.4 Gy	5	EBRT	6-7	1-2x	cur/pall	yes/no	
4x(mix)	12-30	1.8-2.5 Gy	24-54 Gy	yes	5	EBRT	4-10	1-2x	cur/pall	yes/no	
2x(mix)	4 (bid)	7.5-9 Gy	30-36 Gy		1	Brachy	1	1x	pall	yes	

Sarcoma

Nos.	Fx	Dose/fx (Gy)	Total dose (Gy)	Included boost	RT**frequency	RT modality	dHT sessions	dHT frequency**	Treatment intent	Re-RT	Ref
5x	25	2 Gy	50 Gy		5	EBRT, PT	1*, 5-10	1-2x	curativ	no	[33]
3x(mix)	33-38	2-2.15 Gy	70-76Gy		4-5	PT	7-8	1x	2x cur, 1x pall	yes/no	[34,53]

Pancreatic cancer											
**Nos.**	**Fx**	**Dose/fx (Gy)**	**Total dose (Gy)**	**Included boost**	**RT**frequency**	**RT modality**	**dHT sessions**	**dHT frequency****	**Treatment intent**	**Re-RT**	**Ref**
6x	28	2 Gy	56 Gy		5	EBRT	4-6	1x	5x cur; 1x pall	no	[36,50]
2x(mix)	25 -28	1.8 – 2 Gy	50 – 50.4 Gy		5	EBRT	5-6	1x	cur	no	[36,50]

Local tumor recurrence and compression											
**Nos.**	**Fx**	**Dose/fx (Gy)**	**Total dose (Gy)**	**Included boost**	**RT**frequency**	**RT modality**	**dHT sessions**	**dHT frequency****	**Treatment intent**	**Re-RT**	**Ref**
2x	15	2 Gy	30 Gy		5x	EBRT	4-5	1-2x	pall	yes	
3x	13	3 Gy	39 Gy		5x	EBRT	4-5	1-2x	pall	yes/no	
2x	27	2 Gy	54 Gy		5x	EBRT	6-8	1-2x	pall	no	
1x	35	2 Gy	70 Gy		5x	PT	8	1x	pall	yes	
1x	4 (bid)	7 Gy	28 Gy		1x	Brachy	1	1x	pall	yes	
1x	4	7.5 Gy	30 Gy		2x	SBRT	1	1x	pall	yes	
8x(mix)	10-33	1.8-3 Gy	25-59.4 Gy	yes	5x	EBRT, 1x PT	3*, 3-8	1-2x	pall	yes/no	

Painful bony metastasis											
**Nos.**	**Fx**	**Dose/fx (Gy)**	**Total dose (Gy)**	**Included boost**	**RT**frequency**	**RT modality**	**dHT sessions**	**dHT frequency****	**Treatment intent**	**Re-RT**	**Ref**
2x	12	2.5 Gy	30 Gy		5x	EBRT	5	2x	pall	yes	
1x	5	2.5 Gy	12.5 Gy		2x	EBRT	5	2x	pall	yes	
4x(mix)	11-27	2-3 Gy	33 – 54 Gy		5x	EBRT	4-10	1-2x	pall	no	[39]

Abbreviations: bid: bis in die (twice per day), Brachy: HDR-Brachytherapy, dHT: deep hyperthermia, EBRT: external body radiotherapy, Fx: fraction, Gy: Gray, mix: several treatment schedules are depicted together, Nos.: numbers, pall: palliative intention, PT: proton therapy, RT: radiotherapy, re-RT: re-irradiation, Ref: reference, SBRT: stereotactic body radiotherapy, *: HT interrupted, ** times per week.
